# An Intelligent System for Early Recognition of Alzheimer’s Disease Using Neuroimaging

**DOI:** 10.3390/s22030740

**Published:** 2022-01-19

**Authors:** Modupe Odusami, Rytis Maskeliūnas, Robertas Damaševičius

**Affiliations:** 1 Department of Multimedia Engineering, Kaunas University of Technology, 51368 Kaunas, Lithuania; modupe.odusami@ktu.edu (M.O.); rytis.maskeliunas@ktu.lt (R.M.); 2 Department of Software Engineering, Kaunas University of Technology, 51368 Kaunas, Lithuania

**Keywords:** intelligent systems, image processing, expert systems, Alzheimer’s disease, MRI, deep learning, explainability

## Abstract

Alzheimer’s disease (AD) is a neurodegenerative disease that affects brain cells, and mild cognitive impairment (MCI) has been defined as the early phase that describes the onset of AD. Early detection of MCI can be used to save patient brain cells from further damage and direct additional medical treatment to prevent its progression. Lately, the use of deep learning for the early identification of AD has generated a lot of interest. However, one of the limitations of such algorithms is their inability to identify changes in the functional connectivity in the functional brain network of patients with MCI. In this paper, we attempt to elucidate this issue with randomized concatenated deep features obtained from two pre-trained models, which simultaneously learn deep features from brain functional networks from magnetic resonance imaging (MRI) images. We experimented with ResNet18 and DenseNet201 to perform the task of AD multiclass classification. A gradient class activation map was used to mark the discriminating region of the image for the proposed model prediction. Accuracy, precision, and recall were used to assess the performance of the proposed system. The experimental analysis showed that the proposed model was able to achieve 98.86% accuracy, 98.94% precision, and 98.89% recall in multiclass classification. The findings indicate that advanced deep learning with MRI images can be used to classify and predict neurodegenerative brain diseases such as AD.

## 1. Introduction

Alzheimer’s disease (AD) is a brain disease that has become more prevalent over time and it is now the fourth leading cause of mortality in industrialized nations. Memory loss and cognitive decline are the most common signs of AD, and are caused by damage to and death of nerve cells related to memory in the brain area [1]. Mild cognitive impairment (MCI) is a stage that occurs between normal and AD [2]. AD progresses gradually through the prodromal stage of MCI, and finally, to AD dementia. According to studies, people with MCI acquire AD at a rate of 10–15% every year [3]. Early identification of patients with MCI can delay or prevent the progression of the disease from the MCI stage to AD. The morphological differences in the brain lesions in patients with intermediate stages of MCI are very small [3]. Furthermore, they have similar clinical manifestations; thus, to act early in the diagnosis and treatment of AD, the diagnosis and prognosis of MCI disease have been analyzed using magnetic resonance imaging (MRI) studies [4], which can capture alterations in the brain anatomy and function [5]. In recent years, several machine learning algorithms have been used for the early diagnosis of AD using magnetic resonance imaging. A support vector machine (SVM) with particle swarm optimization was applied to the classification of AD and MCI [6]. The authors of [7] suggested the exploration of deep learning in their future studies. The authors of [8,9] reported the use of clustering techniques for clustering AD stages for early detection. The study in [10] revealed that deep learning and SVM produced higher accuracies in the classification of AD stages. However, given the very high dimensions of the input image, SVM may not be advantageous [11]. Deep learning methods have now been proposed for automatic recognition of dementia diseases [12,13] due to their ability to create an effective prediction model; in particular, convolutional neural networks (CNN) have shown great success in the computer-assisted diagnosis of AD and MCI, and are beneficial for accurate classification of AD.

In the latest studies, pre-trained CNNs have proved to be excellent in the automatic diagnosis of cognitive disease from brain MR images. AlexNet [14,15], VGG16 [16,17], VGG11 [18], ResNet-34 [19], ResNet-50 [20], U-Net [21,22], SqueezeNet and InceptionV3 [23], and DenseNet201 are examples of pre-trained deep neural networks that have been effectively used in MRI analysis. Compared to a model that comprises a single network pre-trained on MRI slices, multiple pre-trained networks on a large scale with MRI may gather potentially useful functional and structural information for discriminating the AD stages. An informative feature should keep all the important information from the input image. Comparatively, a model based on multiple pre-trained networks has shown outstanding performance in the classification of AD and MCI classification [24]. Due to the complex structure of MRIs and because the functional changes in the brain in the AD and MCI intermediate classes are closely related, multiple pre-trained networks have been designed to obtain meaningful information randomly from different layers of the pre-trained networks. This information is then combined or concatenated to extract multi-scale information from the input images [25,26]. Concatenation produces a discriminant and appropriate descriptor to further improve the power of the features of the classifier model. For example, the authors of [24] exploit the voting technique to get the required information for classification from the decision of the hybrid model. Although the model extracted the more correlated and diversified features, it could not generalize well on unseen data.

In this paper, we propose a randomized concatenation of deep features approach for automatic discrimination of patients with AD, early MCI (EMCI), late MCI (LMCI), MCI, and cognitively normal (CN) using deep learning architectures to take advantage of random information from MRI brain imaging data. This method was used to develop a definite categorization descriptor. First, each pre-trained model receives the discriminating information for the different classes from the MRI imaging data. A concatenation technique in which two fully connected layers are appended to combine the features learned, then a constant (weight) is added in concatenation. The idea behind the proposed method is that weight can reduce the value of a part of feature maps in concatenation, and then the multiplied convolutional weight will automatically enlarge the useful feature maps.

The primary contributions of this paper may be summarized as follows.

- A hybrid pre-trained CNN model for the early diagnosis of AD.
- A deep feature concatenation method for merging deep features collected from various pre-trained CNNs.
- Weight randomization to lessen the gap between feature maps in the concatenation of fully connected layers.
- Gradient-weighted class activation map to visualize discriminative regions of the image to explain the model’s decision.

The remainder of the paper is structured as follows. Section 2 describes the recent research on the early detection of AD. Section 3 illustrates the proposed hybrid model for diagnosing AD. Section 4 shows the result of the experiment on the ADNI dataset, and the discussion is detailed in Section 5. Section 6 shows the comparison of the model with previous research, while we conclude the study in Section 7.

## 2. Related Work

Several CNN-based techniques that were pre-trained have been utilized for multiclass classification and binary classification for the early detection of AD. The authors of [26] adopted Resnet 152 to obtain a highly discriminative feature representation for the classification of AD stages. A four-way classifier was implemented to classify AD, MCI, LMCI, and CN using ADNI imaging data. The proposed technique resulted in a prediction accuracy of 98.8%. The authors of [27] utilized VGG16-trained learning transfer for the multiclass classification of AD based on four stages (CN, AD, MCI, LMCI). The proposed model gave a testing accuracy of 95.31%. The study [28] employed multiple deep sequence-based models using 3D ResNet18 with data augmentation Resnet18 to extract features for accurate AD classification and achieved a classification accuracy of 96.88%.

In [29], the authors developed a deep learning approach based on modified Resnet18 for the binary classification of AD, including EMCI versus LMCI, AD vs. CN, CN vs. EMCI, CN vs. LMCI, EMCI vs. AD, LMCI vs. AD, MCI vs. EMCI, and MCI vs. EMCI. Their method provided an accuracy of 99.99% for EMCI vs. AD. The authors of [30] used a transfer learning technique for the three-way classification of MRI images using three pre-trained convolutional neural networks (CNN), namely: ResNet-101, ResNet-50, and ResNet-18. The experimental results showed that ResNet-101 gave an accuracy of 98.37%.

The authors of [31] proposed a 2D CNN approach based on ResNet50 with the addition of different batch normalization and activation functions to classify brain slices into three classes: cognitively normal (NC), mild cognitive impairment (MCI), and AD. The proposed model achieved an accuracy of 99.82%. Another study [32] introduced the SegNet-based deep learning method to extract AD-related brain morphological local characteristics, which are required to classify the AD condition. Resnet101 performed a three-class classification, and the results showed that the use of a deep learning technique with a pre-trained model proved highly helpful in improving the performance of the classifier. A 3D CNN was used in [33] to develop a classifier that could discriminate between AD and CN from resting-state fMRI images while achieving the accuracy of 97.77%.

The authors of [34] developed a unique method for diagnosing AD stages using probability-based fusion of various CNN models based on the DenseNet network. The proposed model was able to perform a four-way classification of CN, EMCI, LMCI and AD, on the ADNI dataset. The experimental results showed that the proposed model gave an accuracy of 83.33%. In [35], the authors used a three-dimensional net-121 architecture with a dropout rate of 0.7 for training, using the entire set of data to obtain an accuracy of 87%.

For the diagnosis of AD and MCI, the authors of [36] suggested an ensemble of densely linked 3D convolutional networks in which dense connections were introduced to improve the use of features. The ensemble model achieved an obvious improvement in its accuracy (with a 97.52% accuracy) compared to just using the simple average of the networks’ predictions. The authors of [37] used convolutional network topologies for binary classification using freeze characteristics taken from the ImageNet source data set. The results of the study showed that the VGG architecture gave an accuracy of 99.27% (MCI/AD), 98.89% (AD/CN) and 97.06% (MCI/CN).

The authors of [38] proposed a layer-wise transfer learning method using VGG 19 that segregated between normal control (NC), early mild cognitive impairment (EMCI), late mild cognitive impairment (LMCI) and AD. The authors of [39] suggested a transfer learning technique to reliably categorize brain sMRI slices into three classes: AD, CN and MCI. The authors used a pre-trained VGG16 network as a feature extractor and for transfer learning. The proposed model achieved a classification accuracy of 95.73%.

The authors of [40] proposed an approach for efficient classification of AD using a pre-trained AlexNet network to efficiently extract significant information from the MRI data. Their model achieved a classification accuracy of 98.35%. The authors of [41] used transfer learning to classify images from the OASIS database by fine-tuning a pre-trained AlexNet. The model showed promising results with an accuracy of 92.85% for multiclass classification. Furthermore, the authors of [42] employed a modified AlexNet with parameters to adjust for the classification of four stages of AD. The proposed strategies showed results with an accuracy of 95.70%.

The authors of [43] used fMRI data sets to classify different stages of the disease using the architecture of a CNN AlexNet. The severity of AD was divided into five phases according to the proposed model. Low- to high-level characteristics were learned during classification, resulting in an average accuracy of 97.64%. Their conclusion was that the incorporation of additional pre-trained models and transfer learning could increase classification accuracy. Similarly, the authors of [44] used modified AlexNet, ResNet-18 and GoogLeNet for the classification of brain images of CN, EMCI, MCLI, LMCI, and AD. The experimental results also showed that ResNet18 performed better.

In another study, the authors of [45] utilized different pre-trained models using a fine-tuned approach to transfer learning in the ADNI dataset for three-way AD classification (AD, MCI, and NC). The experimental results showed that DenseNet outperformed the others, achieving a maximal average accuracy of 99.05%. The authors of [46] performed an experimental analysis of some deep neural networks for the classification of AD. DenseNet-121 was found to be better than all other models used for the analysis with a classification accuracy of 90.22%.

## 3. Proposed Randomized Concatenated Deep Features Approach

**Figure 1:**
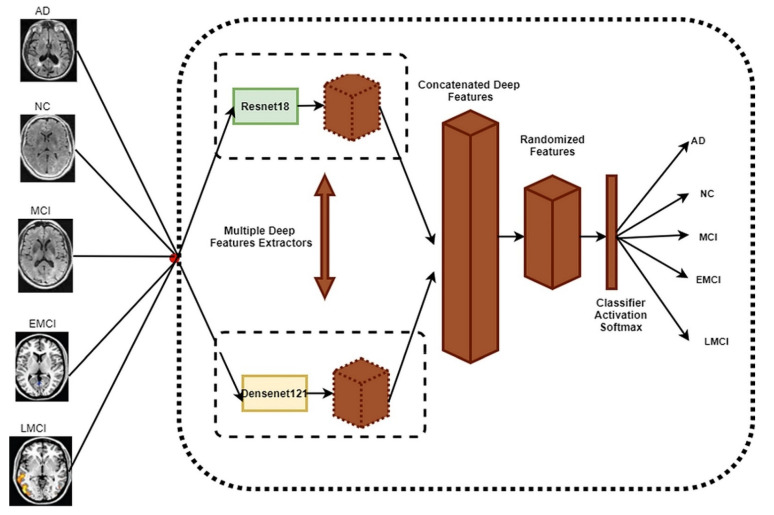
The main framework for the proposed method.

As previously stated, the suggested technique seeks to provide an accurate diagnosis of Alzheimer’s disease by weight-randomizing concatenated deep features taken from the ResNet18 and DenseNet121 networks. Figure 1 shows the pipeline of the proposed randomized concatenated deep features-based classification system to identify patients with AD, MCI, EMCI, LMCI, and NC clinical status using MRI neuroimaging data.

### 3.1. Dataset

**Figure 2:**
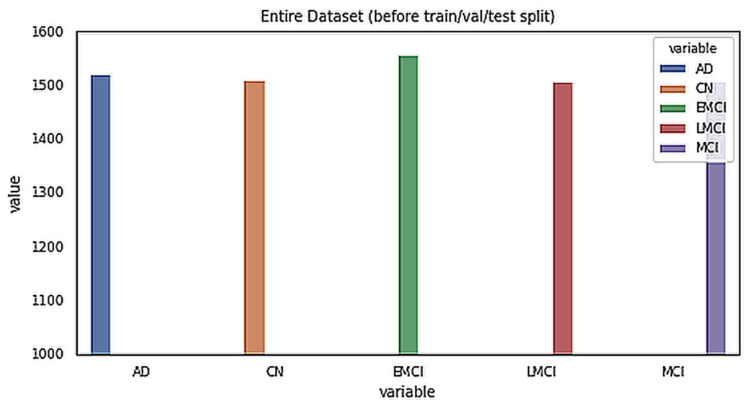
Class distribution of the MRI dataset.

**Figure 3:**
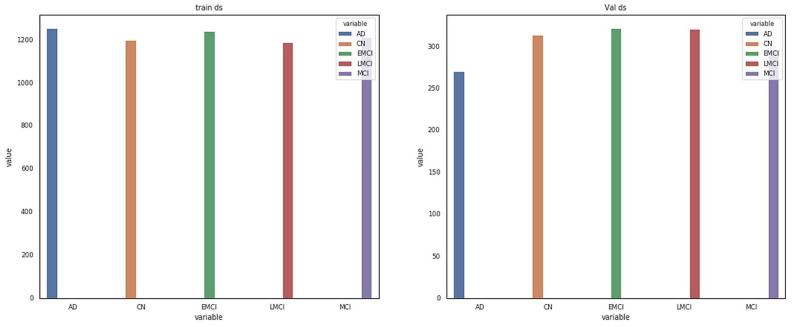
Details of dataset splitting per class.

The data for this study were obtained from the ADNI database. ADNI is a long-term research project aimed at developing clinical, imaging, genetic, and biochemical indicators for the early diagnosis and tracking of Alzheimer’s disease. There are 138 MRI scans in the dataset, with 25 CN, 25 SMC, 25 EMCI, 25 LMCI, 13 MCI, and 25 AD scans [18]. The participants are above the age of 71, and each has been diagnosed with Alzheimer’s disease and assigned to one of these phases according to their cognitive test results [18]. Figure 2 shows the class distribution of the MRI dataset. A total of 7509 processed MRI images were evaluated from the ADNI database and the data split utility (random split) in PyTorch divided the data into 5256 and 2253 images for training and validation, respectively. Details of the data splitting are given in Figure 3. A new set of samples were extracted from subjects that were separate from the subjects used for training and validation to test the model for generalizability.

### 3.2. Deep Feature Extraction

The ResNet18 and DenseNet121 deep CNN architectures were used in the feature extraction procedure from MRI images. In CNN designs, multiple layers are used to extract features. The offered layers include convolutional pooling, batch normalization, rectified linear unit (ReLU), SoftMax, and fully connected (FC) [47]. The FC layer was kept the same for all models. The convolutional layer was made up of several weights $H^{l}$ kernels for each layer y to take the input $X^{y-1}$ and extract the local characteristics. Both Resnet18 and DenseNet121 were set with weights that had been pre-trained using natural photos from ImageNet [48]. Then, we ran each model one time on our training and validation images to extract the deep features.

### 3.3. Concatenation of Deep Features

Concatenation of the recovered deep features is an effective approach to combine multiple characteristics to improve the classification process [49]. In this study, the concatenation procedure was achieved by extracting characteristics from images using Resnet18 and DenseNet121 features. The high-level features of the entire connected layers of both Resnet18 and Densenet21 were linked into a single vector to form the classification descriptor as shown in Equation (1).

(1) $$Feature\;Descriptor=F^{\left(Resnet-18\right)}\cup^{\;}F^{\left(Densenet-121\right)}$$

### 3.4. Weight Randomization and Classification

Combining information by concatenating or adding leads to different information being mixed in the fusion layer. However, some of this information may be useless, and as such, narrowing the gap among the fused layers can result in better classification accuracy. In this study, an inbuilt weight initialization in PyTorch was implemented, including a uniform Kaiming distribution [50,51] and Xavier (Glorot). The Kaiming weight initialization was created to perform non-symmetrical activation functions such as ReLUs, while the Xavier initialization was designed for layers with sigmoid activators. Each layer’s weights are created using a normal distribution. The resulting tensor using the Kaiming weight will have values sampled from N (−bound, bound), where bound is described in Equation (2).

(2) $$bound=gain\;\cdot \sqrt{\frac{3}{fan\_mode}}$$

The resulting tensor using Xavier will have values sampled from U(−a, a) where a is described in Equation (3).

(3) $$a=gain\;\cdot \sqrt{\frac{6}{fan_{in}+fan\_out}}$$

The following parameters depicted in Table 1 are considered in the Kaiming weight initialization and the Xavier weight initialization.

The last description of the feature determines whether the input image is classified as MCI or LMCI or EMCI or AD or CN. The fully connected layer turns the input data into a 1D vector, and the SoftMax layer computes the five class scores.

### 3.5. Gradient-Weighted Class Activation Map (Grad-CAM)

Grad-CAM is the generalization of the class activation map (CAM), which locates the discriminative region(s) for a CNN prediction by computing class activation maps with gradient information. Grad-CAM creates a map of the working class by incorporating gradient data into the final decision layers. Grad-CAM weighs the 2D activations with the average gradient. It helps to understand what the network sees, and which neurons are firing in a particular layer, given an input image [51]. The last class gradient related to a channel is used to measure all channels, following the final convolution layer to create a localization map showing the critical locations in an image that have a significant impact on the model forecast. To obtain the class discrimination localization map, the class score gradient was calculated relative to the feature maps of the convolutional layer [52]. Then, to obtain the key weights of the neuron, we took a global average of these gradients as described in Equation (4).

(4) $$\propto_{u}^{v}=\frac{1}{Y}\;\overset{s}{\underset{i=0}{\sum }}\;\overset{t}{\underset{j=0}{\sum }}\frac{\partial w^{v}}{\partial X_{ij}^{u}}$$

where s is width for any class, v is height for any class, $\partial w^{v}$ is gradient for the class score, $X^{U}$ are feature maps, and $\propto_{u}^{v}$ are neuron weights.

Finally, the Grad-CAM map was obtained by linearly combining the weights with the activation map of ReLU.

### 3.6. Implementation Details

Our proposed study was created on the NVIDIA Corporation Tu116 (Geforce GTX 1660) GPU using Python 3.6 with the Pytorch package. The proposed study was evaluated on the prepared dataset using the random split approach, and the details of the data split can be found in Table 1. The images of the dataset were resized to 224 × 224 pixels, and a batch size of 10 was kept due to memory usage with the number of epochs, which was 10. The number of loader worker processes was set to 4.

For optimization of the hyperparameters of the deep learning model, we used the AutoKeras 1.0.8 library, which optimizes both the architecture and hyperparameters as guided by the Bayesian optimization to select the best hyperparameter values [53]. Based on the hyperparameter optimization stage, the learning rate was fixed to 0.0001 for the training of the proposed model. A dropout of 0.4 and weight decay of 0.002 were also used to fit the proposed model for the purpose of fine-tuning.

**Figure 4:**
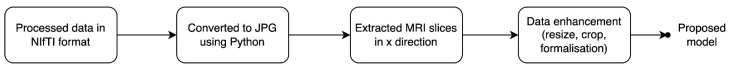
Detailed workflow of data used in the proposed model.

**Figure 5:**
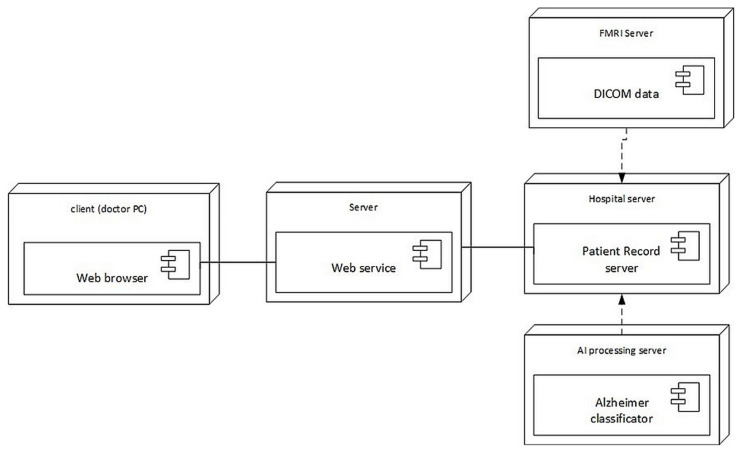
Deployment of the developed system architecture.

The architecture of the prototype system developed for AD diagnostics is presented in Figure 4, while its deployment is shown in Figure 5. The doctor uses his/her personal computer to access the patient’s records on the hospital server via a dedicated web service. The hospital server retrieves the patient’s brain MRI images from the MRI server, which are stored in NIfTI (Neuroimaging Informatics Technology Initiative) format. The diagnostic decision is taken by the Alzheimer’s disease classifier implementing the approach described in this paper. The decision is supported by Grad-CAM attention images that explain the suggested decision.

## 4. Experimental Results

**Figure 6:**
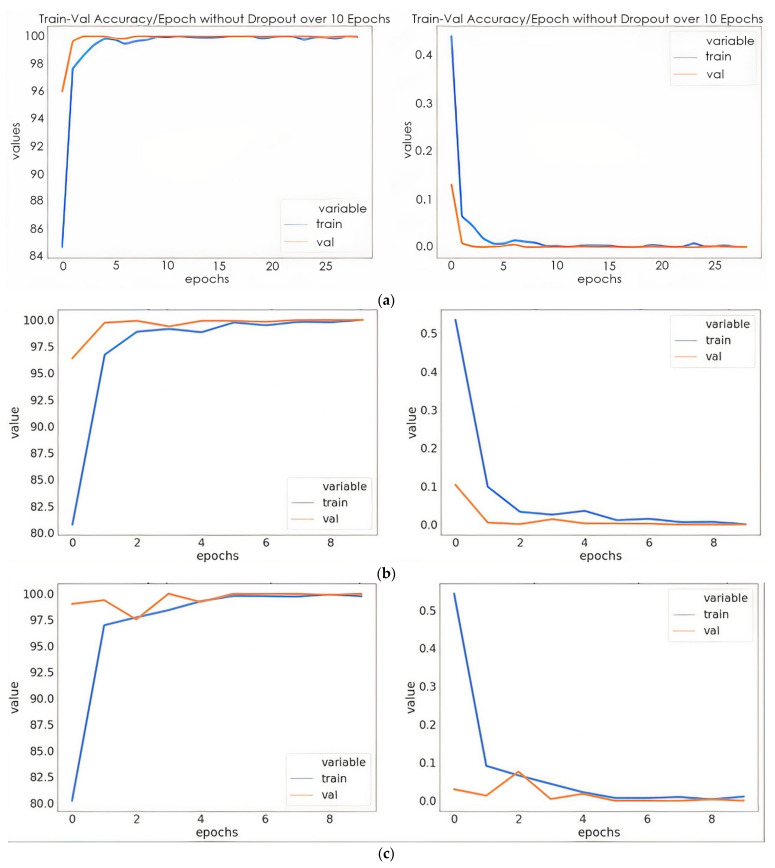
Training/validation accuracies and training loss/validation loss: (**a**) model without dropout; (**b**) mode with dropout; (**c**) model with dropout and weight decay discussions.

We provide the experimental results for one five-way benchmark multiclass classification problem [18], one four-way multiclass classification problem [33], and one three-way classification problem [45]. The suggested model’s training efficiency was evaluated in terms of important parameters, i.e., training accuracy, validation accuracy, training loss, and validation loss at different epochs without dropout, with dropout, with dropout and weight decay. Learning rates of 0.0001 and 0.0003 were optimized with stochastic gradient descent (SGD) and adaptive moment estimation (ADAM). The proposed model performed better when using a learning rate of 0.0001 with the SGD optimizer. These parameters are calculated to estimate the trained models with a learning rate of 0.0001 optimized with SGD. These parameters are calculated to estimate the overfitting of the trained models. The graphs of the training loss/validation accuracy and training accuracy/validation accuracy of the proposed model with a random split are depicted in Figure 6. Furthermore, a confusion matrix was generated for the proposed model to quantify the performance metrics, i.e., precision, recall, F1 score, and accuracy. The results of the parameters with the five-way multiclass, the four-way multiclass, and the three-way multiclass problem using the Kaiming and Xaiver weight initialization are presented in Table 2 and Table 3, respectively. Table 4 shows the results of the suggested model on the test dataset, while Table 5 shows the results of the proposed model on test data from different subjects. Each of the classes consists of 20 slices each.

**Figure 7:**
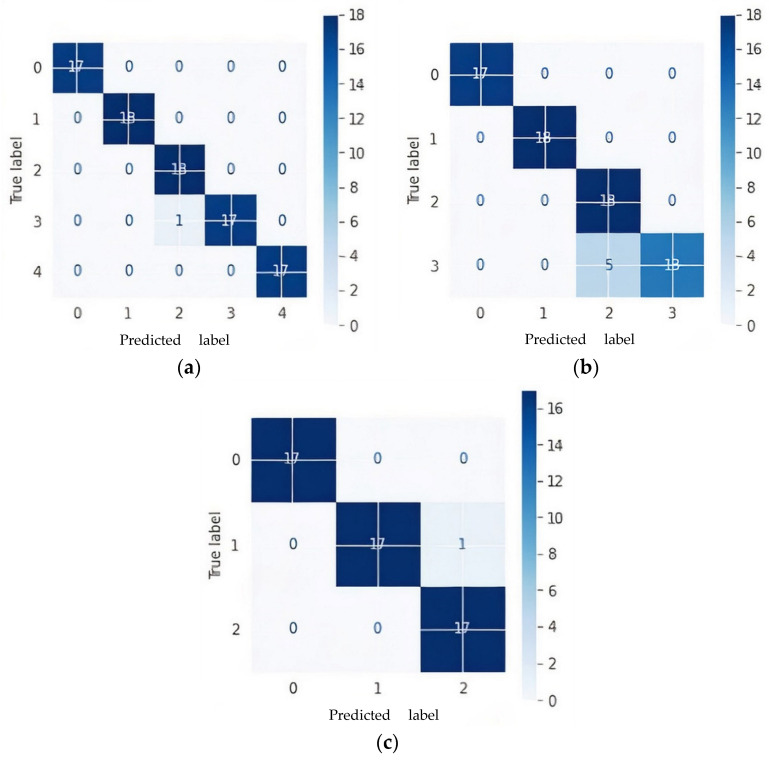
Confusion matrices of proposed model based on Kaiming weight initialization on test data: (**a**) 5-way multiclass; (**b**) 4-way multiclass; (**c**) 3-way multiclass.

The confusion matrix was generated using a test dataset taking samples from one subject that was not used for training. The confusion matrix for a five-way multiclass, four-way multiclass, and three-way multiclass is represented in Figure 7 and 0, 1, 2, 3, and 4 labels are represented as AD, CN, EMCI, LMCI, and MCI, respectively.

**Figure 8:**
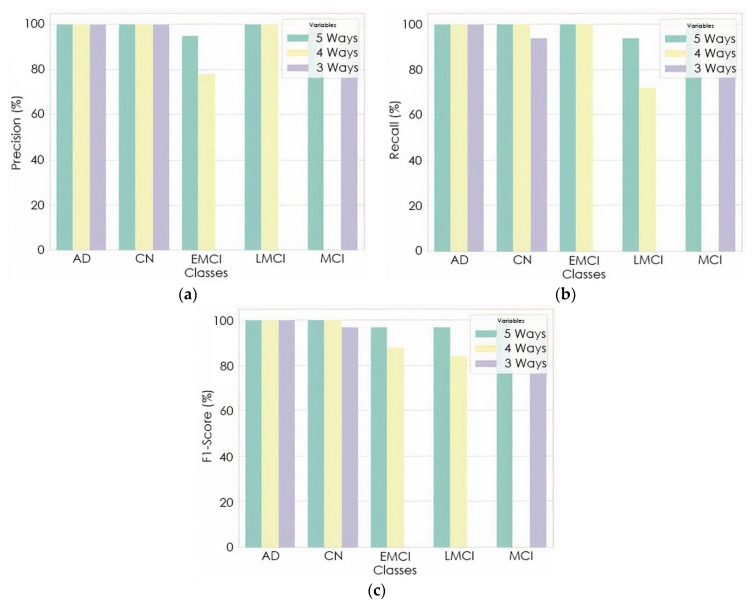
Per class classification report: (**a**) precision; (**b**) recall; (**c**) F1-Score.

The proposed model gave an average precision and recall of 98.94% and 98.89%, 89.61% and 88.89%, 98.14%, 98.14% for the five-way, four-way, and three-way multiclass, respectively. The per class classification report of the proposed model based on precision, recall, and F1-score is detailed in Figure 8. Furthermore, the proposed model was checked to see if the predicted label matched the actual label by examining the number of images predicted correctly and incorrectly.

**Figure 9:**
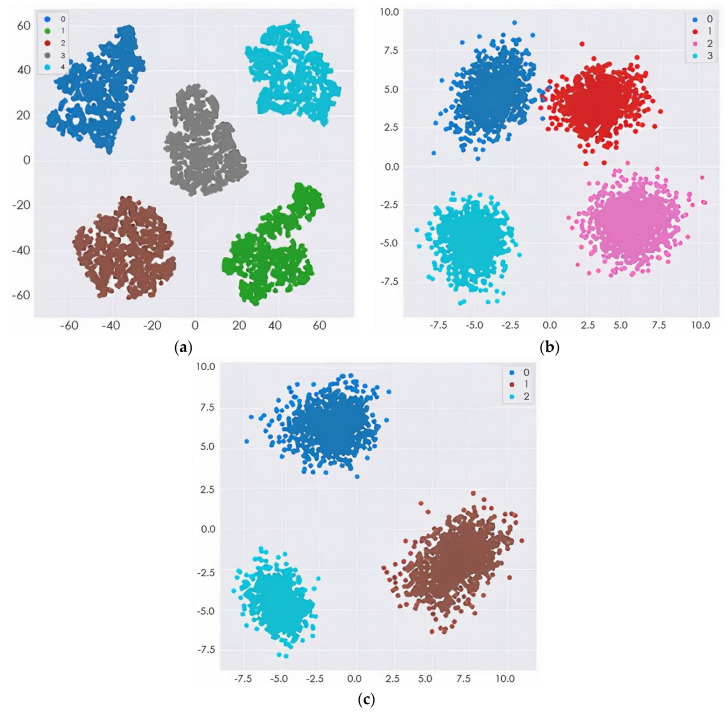
The t-SNE projection visualization of several MRI features: (**a**) 5-way multiclass classification; (**b**) 4-way multiclass classification; (**c**) 3-way multiclass classification.

To verify the efficacy of our proposed technique, the representation of the output data from the model before classification was featured in the form of cluster figures by the t-SNE projection, as shown in Figure 9 for the four-way multiclass and three-way multiclass classification. This was achieved by reducing the dimensional output layer down to five dimensions, four dimensions, and three dimensions for five-way, four-way, and three-way classification, respectively.

**Figure 10:**
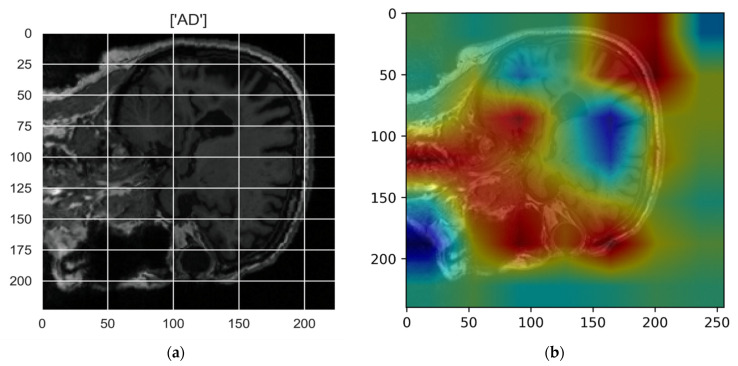
Grad-CAM explanation for the prediction of class AD: (**a**) MRI image, (**b**) Grad-CAM attention map.

**Figure 11:**
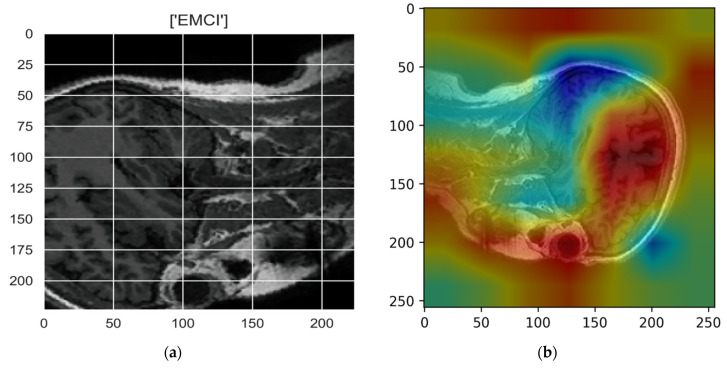
Grad-CAM explanation for the prediction of class EMCI: (**a**) MRI image, (**b**) Grad-CAM attention map.

**Figure 12:**
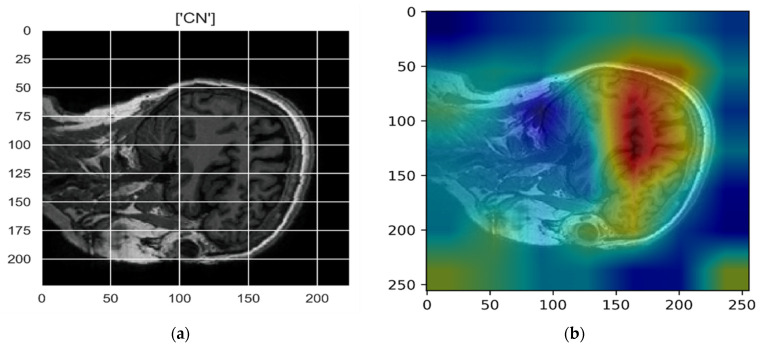
Grad-CAM explanation for the prediction of class CN: (**a**) MRI image, (**b**) Grad-CAM attention map.

**Figure 13:**
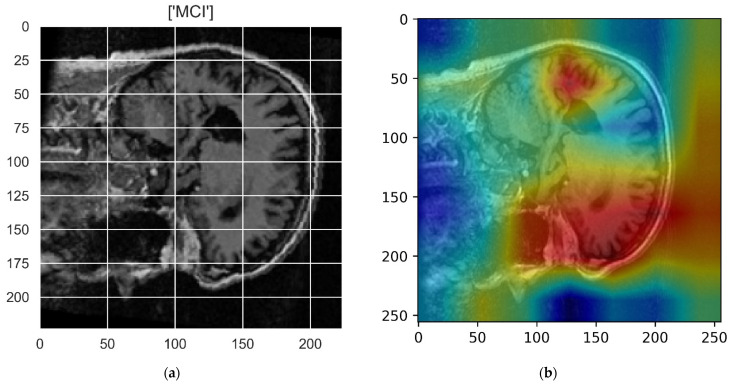
Grad-CAM explanation for the prediction of class MCI: (**a**) MRI image, (**b**) Grad-CAM attention map.

**Figure 14:**
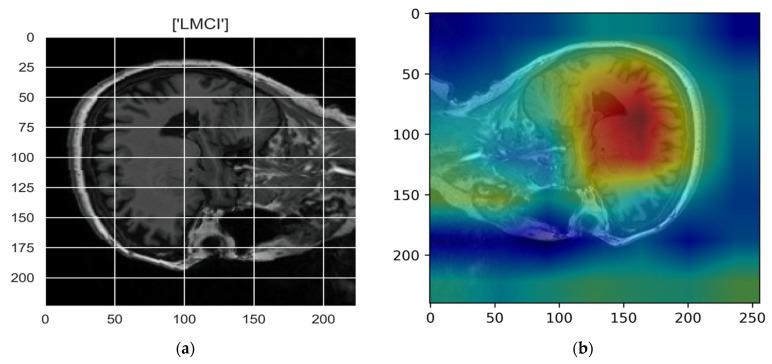
Grad-CAM explanation for the prediction of class LMCI: (**a**) MRI image, (**b**) Grad-CAM attention map.

Figure 10, Figure 11, Figure 12, Figure 13 and Figure 14 show the result of the Grad-CAM on the predicted classes AD, EMCI, CN, MCI, and LMCI respectively.

## 5. Discussion

The proposed model has minimal training and validation loss and exhibits the best training and validation accuracy with dropout (0.4) and a weight decay of 0.02. In Figure 6a, the training and validation accuracy of the proposed model without dropout clearly show that the training accuracy was steady at 6 epochs. This indicates that the model was no longer learning. Likewise, the validation accuracy remained the same after 5 epochs. Although the model was able to fit adequately with the use of a 0.4 dropout, the accuracy was lower than that of the model with a dropout of 0.4 and weight decay of 0.002. The overfitting was reduced to minimal with the use of dropout and weight decay. However, the validation accuracy was slightly higher than the training accuracy with a very close margin as the number of epochs increased. In Table 2 and Table 3, the proposed model provided the best training and validation accuracy at 5 epochs for all the ways of multiclass classification. As shown in Table 4, the proposed model based on the initialization of the Kaiming weight produced a testing precision with a three-way multiclass classification accuracy of 100%, a four-way multiclass classification accuracy of 95.83%, and a five-way multiclass classification accuracy of 98.86%. It was evident from the results that the proposed model accurately discriminates between three-way multiclass (AD/MCI/CN) and five-way multiclass (AD/CN/LMCI/EMCI/MCI) classifications of Alzheimer’s disease. However, for the purpose of this study, more emphasis was on the five-way multiclass classification. Table 4 shows that for the Kaiming weight initialization technique, the three different multiclass methods demonstrate the highest test accuracies. Therefore, Kaiming weight initialization was set for this study. As we can see in Table 4, for all classification problems, the test accuracy of the proposed model using the Kaiming weight initialization is superior to the Xavier weight initialization technique. The result of the generalization of the proposed model on different test data is shown in Table 5 with a standard deviation of 0.42, 0.50 and 0.42 for five-way, four-way, and three-ways multiclass classification, respectively.

Figure 6 shows that one subject of LMCI was misclassified as EMCI as in the case of five multiclass classifications while five LMCI were incorrectly diagnosed as EMCI in four multiclass classifications. Although LMCI was found to be very close to being diagnosed as AD, from the confusion matrixes in Figure 6a,b, it was seen as EMCI. This is because a single modality cannot capture more disparate differences between EMCI and LMCI. One CN was misclassified as MCI in both five-way and three-way multiclass, which was an indication of an effective model because in medical diagnosis, it is preferred to screen a person as diseased than to eliminate a diseased person by falsely predicting a negative.

As shown in Figure 7, a precision of 95% and 73% was achieved to distinguish EMCI from other classes in five-ways and four-ways multiclass, respectively, while a precision of 100% and 86% was also achieved to distinguish LMCI from other classes in five-way and four-way, respectively. The proposed model also achieved a precision of 94% and 94% to differentiate MCI from other classes in five-way and three-way multiclass classification, respectively. From the per class classification result in Figure 7, five-way multiclass had the highest recall of 100% and 94% in differentiating EMCI and LMCI, respectively, from other classes.

The proposed model was trained to be incredibly confident in its predictions, as an image with a confidence score of 0.687 had the predicted intent of being a solid subject for the predicted class. This result also showed that if the features of the different classes (EMCI and LMCI) could be more represented, the misclassification error would be greatly reduced, thus increasing the classification accuracy. The confidence in the predicted label was very high because some of the numerical properties provided by the initialization technique were not kept when the training process updated the weight values. The confidence level of the proposed model for the incorrectly classified image was 0.602. The designated classes of each multiclass classification look well separated, as depicted in Figure 8. This could improve the model classification performance.

In the Grad-CAM maps in Figure 10 and Figure 11, the red regions highlight the most important discriminative regions, and other colored regions are less important. In MRI images with a sagittal plane view, the model focuses on the vermis of cerebellum [54], and the fourth ventricle areas of the posterior and anterior lobes [55] are involved in the prediction of the AD class. For the EMCI prediction, white matter hyperintensities (WMH) are found to be associated with EMCI class prediction [56]. In Figure 12 and Figure 13, the model focused on the internal cerebra vein to identify the pattern for the prediction of CN and MCI, as cerebral basal vein dilation is related to the volume of the white matter hippocampus (WMH) [57].

## 6. Comparison with Existing Methods

To our knowledge, our approach is the first to have a concatenated randomized output from two pre-trained models for the early diagnosis of Alzheimer’s disease based on a multiclass classification of five-ways, four-ways, and three-ways. To assess our method, we compared the proposed technique to similar studies that used the same parameters, as shown in Table 6. It can be clearly seen that the proposed method yielded the best results in regard to all metrics. The neuroimaging data of all these comparative studies are based on the ADNI website, and their methods are confined to pre-trained networks.

As described in Section 2, the studies described in [18,33,58] utilized deep learning using the transfer learning method for the early diagnosis of Alzheimer’s disease. As shown in Table 6, the multiclass classification of the proposed method in the five-way multiclass classification outperformed the results of [18], achieving an accuracy of 98.86%, which is 0.98% higher than that achieved by the study in [18], and the classification accuracy in the four-way multiclass classification is much higher than that of the study in [33], demonstrating the effectiveness of our model. Additionally, in our comparative analysis, we discovered that our model performed better than the study in [58], as our model achieved a classification accuracy of 98.21% and a precision of 98.14%.

## 7. Conclusions

In this study, we adopted two pre-trained models to learn features simultaneously from MRI images, and the learned features were concatenated for AD classification. The concatenation of features amounted to distant or irregular information in fully connected layers during the classification process, and we hypothesized that adopting weight randomization would reduce the gap between feature maps. We tested our hypothesis by performing detailed experiments using brain MRI images from the ADNI dataset, which serves as a benchmark, where 25 subjects were used from each of the five categories of AD, MCI, EMCI, LMCI, and NC (a total of 125 subjects). We investigated the effectiveness of different weight randomization in our application by using Kaiming weight initialization and Xavier weight initialization in our model, and we presented in-depth results and their relation to the number of classes for multiclass classification based on precision. Our results showed that the MRI features concatenated by our proposed model improve the discrimination between classes in five-way multiclass classification compared to four-way multiclass classification.

Finally, our findings were compared to four other state-of-the-art methodologies, where our proposed strategy outperformed them by a significant margin and resulting in a 0.98% and 0.06% improvement in the accuracy of AD vs. CN vs. EMCI vs. LMCI vs. MCI and AD vs. CN vs. EMCI vs. LMCI classification problems, respectively. Likewise, the model achieved an increase of 0.84% and 1.38% in precision for AD vs. CN vs. EMCI vs. LMCI vs. MCI and AD vs. CN vs. EMCI vs. LMCI multiclass classification, respectively. Although the weight mechanism has been proven to be an effective strategy to collect information from feature maps [17], the high classification accuracy of the test data obtained in this paper proved that the weight mechanism can act as a good strategy to automatically enlarge useful feature maps for clinical diagnosis.

There were some limitations in the present study. A multimodal input should be further considered to enhance the capture of more disparate differences between EMCI and LMCI. In addition, a base domain model learned from brain images is possibly more appropriate to the target domain. A large dataset is also required to drastically reduce overfitting. Tackling these limitations in future work will continue to improve the robustness of the recommended approach.

## Figures and Tables

**Table 1:** Parameters for Kaiming and Xavier weight initialization.

Parameters	Description
Tensor	*n*-dimensional torch.Tensor
*a*	Negative slope of the rectifier
mode	“Fan _in” conserves the degree of the variance of the weights in the forward pass
nonlinearity	The non-linear function (nn.functional name)

**Table 2:** The training performance of the proposed model on various multiclass classifications utilizing Kaiming weight initialization.

Ways of Multiclass	Epochs	Training Accuracy (%)	Validation Accuracy (%)	Training Loss	Validation Loss
5 Ways	1	54.93	91.76	1.09	0.32
	2	87.99	98.85	0.33	0.08
	3	96.11	99.87	0.14	0.02
	4	97.97	99.78	0.08	0.01
	5	98.97	99.91	0.06	0.01
	6	99.60	99.10	0.04	0.09
4 Ways	1	50.59	89.21	1.17	0.49
	2	86.86	98.89	0.42	0.12
	3	95.68	99.56	0.17	0.04
	4	96.99	99.61	0.10	0.02
	5	97.91	99.78	0.07	0.01
	6	99.30	98.90	0.05	0.16
3 Ways	1	68.26	93.15	0.76	0.25
	2	89.92	99.26	0.28	0.07
	3	95.36	99.78	0.14	0.03
	4	97.17	99.85	0.09	0.01
	5	97.90	99.93	0.07	0.01
	6	98.50	98.70	0.04	0.16

**Table 3:** The proposed model’s training performance on various multiclass classifications utilizing Xaiver weight initialization.

Ways of Multiclass	Epochs	Training Accuracy (%)	Validation Accuracy (%)	Training Loss	Validation Loss
5 Ways	1	51.20	81.68	1.22	0.67
	2	81.22	96.54	0.59	0.22
	3	93.53	99.07	0.25	0.08
	4	97.32	99.51	0.13	0.04
	5	98.61	99.69	0.08	0.02
	6	99.30	99.73	0.05	0.02
4 Ways	1	44.88	78.63	1.25	0.77
	2	79.29	95.08	0.64	0.28
	3	92.96	99.34	0.28	0.09
	4	96.75	99.83	0.14	0.04
	5	98.29	99.94	0.09	0.02
	6	98.84	99.98	0.06	0.01
3 Ways	1	62.12	86.16	0.85	0.39
	2	85.56	96.35	0.40	0.15
	3	93.26	99.18	0.22	0.07
	4	96.63	99.85	0.13	0.03
	5	97.33	99.78	0.09	0.02
	6	98.31	99.85	0.07	0.01

**Table 4:** Test accuracy and test loss of the proposed model based on Xaiver and Kaiming weight initialization.

Weight Initialization	Ways of Multiclass	Test Accuracy (%)	Test Loss
Kaiming	5 ways	98.86	0.05
	4 ways	93.06	0.14
	3 ways	98.21	0.06
Xaiver	5 ways	87.50	0.43
	4 ways	88.89	0.24
	3 ways	96.21	0.04

**Table 5:** Results of the proposed model for test data with different subjects.

Ways of Multiclass	Accuracy (%) First Test Sample	Accuracy (%) Second Test Sample	Accuracy (%) Third Test Sample	Accuracy (%) Fourth Test Sample	Standard Deviation
5 ways	98.86	98.07	98.98	98.90	0.42
4 ways	93.06	93.02	94.10	93.20	0.50
3 ways	98.21	98.40	98.04	99.01	0.42

**Table 6:** Classification performance comparison.

Authors	Methodology	Multiclass	Accuracy (%)	Precision (%)	Recall (%)
Ramzan et al., (2019) [18]	Resnet 18 (Finetuning)	5 Ways AD/CN/EMCI/LMCI/MCI	97.88	98.10	97.89
Parmar et al., (2020) [33]	3D CNN	4 Ways AD/CN/EMCI/LMCI	93.00	93.18	-
Puete-Castro et al., (2020) [58]	Resnet18 and SVM	3 Ways AD/CN/MCI	78.72	68.96	58.66
Proposed	Resnet18 and DenseNet121 with Randomized weight	5 Ways AD/CN/EMCI/LMCI/MCI	98.86	98.94	98.89
Proposed		4 Ways AD/CN/EMCI/LMCI	93.06	94.56	93.05
Proposed		3 Ways AD/CN/MCI	98.21	98.14	98.14

## Data Availability

The ADNI database is available from http://adni.loni.usc.edu/ (accessed 23 December 2021).
